# Intracochlear drug delivery in combination with cochlear implants: Current aspects

**DOI:** 10.1007/s00106-016-0285-9

**Published:** 2016-12-08

**Authors:** S. K. Plontke, G. Götze, T. Rahne, A. Liebau

**Affiliations:** Department of Otorhinolaryngology, Head & Neck Surgery, University Hospital, Martin Luther University Halle-Wittenberg, Halle (Saale), Germany

**Keywords:** Inner ear, Cochlea, Cochlear implant, Drug delivery, Impedance

## Abstract

Local drug application to the inner ear offers a number of advantages over systemic delivery. Local drug therapy currently encompasses extracochlear administration (i. e., through intratympanic injection), intracochlear administration (particularly for gene and stem cell therapy), as well as various combinations with auditory neurosensory prostheses, either evaluated in preclinical or clinical studies, or off-label. To improve rehabilitation with cochlear implants (CI), one focus is the development of drug-releasing electrode carriers, e. g., for delivery of glucocorticosteroids, antiapoptotic substances, or neurotrophins to the inner ear. The performance of cochlear implants may thus be improved by protecting neuronal structures from insertion trauma, reducing fibrosis in the inner ear, and by stimulating growth of neuronal structures in the direction of the electrodes. Controlled drug release after extracochlear or intracochlear application in conjunction with a CI can also be achieved by use of a biocompatible, resorbable controlled-release drug-delivery system. Two case reports for intracochlear controlled release drug delivery in combination with cochlear implants are presented. In order to treat progressive reduction in speech discrimination and increased impedance, two cochlear implant patients successfully underwent intracochlear placement of a biocompatible, resorbable drug-delivery system for controlled release of dexamethasone. The drug levels reached in inner ear fluids after different types of local drug application strategies can be calculated using a computer model. The intracochlear drug concentrations calculated in this way were compared for different dexamethasone application strategies.

## Background

During the past 25 years, local drug application for therapy of diseases of the inner ear has experienced an increasing interest. The application encompasses:

1. Extracochlear (intratympanic) drug application to the diseased, but intact, inner ear with the objective of protection before exposure (e. g., to a trauma or to substances damaging the inner ear) or after exposure for therapeutic intervention [31, 56].
2. Intracochlear application for drug-, cell-, or gene-based therapy of diseases of the inner ear aiming at regeneration of inner ear structures [14, 35, 57].
3. Extra- and intracochlear application in combination with passive middle ear implants, e. g. in the context of stapes surgery [28].
4. Extracochlear and intracochlear application in combination with auditory prosthesis aiming at improved safety and function of auditory implants [2, 21].

While extracochlear (intratympanic) drug application is already widely used [60], other procedures (2–4 from above) are applied only in individual cases in clinical practice (off-label) or they are the topic of intensive preclinical and translational research [9, 12, 25, 39].

Combinations of the different aforementioned therapeutic strategies are possible, for example, combination of preoperative systemic application, preoperative, extracochlear (intratympanic) application, additional intracochlear application during cochlear implantation, and also postoperative local drug application with the cochlear implant (CI) in place (see case reports later).

## Advantages of local drug delivery to the inner ear

Local application of drugs to the inner ear has many advantages over systemic application (Table 1).

**Table 1:** Advantages of local drug delivery over systemic drug application

– Bypassing of the blood–brain barrier (the target organ is directly reached)
– Higher drug concentration in the inner ear
– Avoiding “first-pass” effects
– Reduction of adverse systemic effects
– Lower drug doses are necessary

This means that local drug application to the inner ear has advantages especially for drugs with:

1. A small therapeutic range
2. Large “first-pass” effects
3. Relevant side effects outside the ear
4. Very expensive pharmaceuticals.

This is especially true, e. g., for neurotransmitters and neurotransmitter antagonists, peptides, viral and nonviral gene transfer, and cell-based therapy.

The indications for drug application in combination with auditory prostheses are generally the same as for auditory prostheses alone (CI, auditory brainstem implant, penetrating auditory brainstem implant, and auditory midbrain implant) [17, 47].

Specific objectives of additional drug application (“drug–device combinations”) in combination with cochlear implantations are summarized in Table 2.

**Table 2:** Objectives of drug application in combination with cochlear implants (“drug–device combinations”). (Modified after Hendricks et al. [17])

– General reduction of insertion trauma
– Reduction of immune reaction
– Reduction of infection
– Reduction of loss of auditory neurons and spiral ganglion cells
– Reduction of fibrosis and ossification
– Reduction of stimulation of nonauditory neural structures
– Reduction of channel interaction
– Improvement of the frequency spectrum, resolution, and the dynamic range of auditory implants

However, currently most of the questions related to local drug delivery in combination with cochlear implants are still open (Table 3).

**Table 3:** Open questions related to drug delivery in combination with cochlear implants

– Which drugs are useful?
– Which strategy of delivery is appropriate: systemic, locally intratympanic, locally intracochlear, or directly via the implant?
– Which time of application is reasonable in the context of extracochlear and systemic therapy: before, during, or after CI implantation?
– Which formulation and which carrier should be used?
– Which delivery system should be applied?
– Which dose should be applied?
– What are the pharmacokinetic characteristics?
– What is the additional benefit of additional drug application?
– What is the risk–benefit ratio?

## Aspects of pharmacokinetics

Rational pharmacotherapy of the inner ear by means of local drug delivery requires specific knowledge of the pharmacokinetics of the inner ear. From a pharmacokinetic point of view, the inner ear is a multicompartment model with almost stationary fluids [27, 50].

The inner ear is a multicompartment model with almost stationary fluids

Numerous aspects of the individual pharmacokinetic processes (liberation, adsorption, distribution, metabolism, and elimination: LADME) have been intensively investigated by our and other groups during the past few years. Nonetheless, many general aspects of pharmacokinetics of the inner ear are still unknown.

Important pharmacokinetic parameters for extra- as well as intracochlear application of drugs (e. g., uptake and elimination) are only known for a view substances. For *elimination,* for example, it was shown that the elimination half-time for dexamethasone from the inner ear of guinea pigs was only around 22.5 min (varying for different parts of the inner ear). Even allowing for a tolerance for the exact value of the elimination half-time, this observation shows that substances for which a longer presence of the drug in the inner ear is desired should be applied continuously either via pump or another application systems with continuous or delayed, controlled release of the substance [23, 26, 33, 41, 49].

Regarding *adsorption* of substances it was demonstrated that intracochlear application of dexamethasone phosphate by injection through the round window membrane leads to a lower variability of the intracochlear concentration, to an increased absolute perilymph concentration, and to a more uniform distribution of the substance in scala tympani [15]. Losses of the substance due to leaks in the round window membrane, however, must be taken into consideration, which occur in the context of injections through the round window membrane. These losses may be reduced by using “sealing” material such as biopolymer gels or biocompatible tissue glue [43].

## Drug-delivery devices

**Fig. 1:**
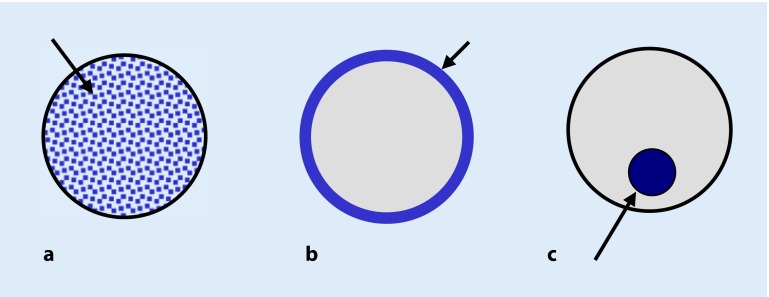
Possibilities of drug delivery with cochlear implant electrodes. **a** Incorporation in the material of the electrode carrier; **b** coating of the electrode carrier; **c** delivery channel and pump

Different options are available for releasing substances by the electrode carrier into the cochlea (Fig. 1).

- The substances may be incorporated in the CI electrode carrier itself.
- The electrode carrier may be coated with the substance.
- The electrode carrier can be equipped with a delivery channel, which is then connected to a drug reservoir or a pump system [4, 18, 24].

Shepherd and Xu suggested a multichannel scala tympani electrode with an application channel for chronic intracochlear infusion [55]. Paasche et al. modified a perimodiolar electrode so that the existing channel of the stiletto required for insertion of the electrode carrier is used for drug delivery [38]. Recent studies focus mainly on coating and incorporation of the substance because a channel application system implies that for the duration of therapy a permanent access to the inner ear is present, which may be associated with a higher risk of infection.

## Drugs delivered in combination with cochlear implantation

### Neurotrophins

The research initially focused on the application of neurotrophins aiming at avoiding or reducing degeneration of spiral ganglion neurons and inducing growth of afferent nerve fibers toward the CI electrodes [16, 48, 64].

Rejali and coworkers coated CI electrodes with fibroblasts. The fibroblast cells were transduced via a viral vector with a BDNF gene. The BDNF-secreting cells were attached to the CI electrode by means of an agarose gel and the electrodes were implanted into the scala tympani of guinea pigs. In comparison with the control group, the authors found after 48 days that in the group with BDNF-releasing electrodes significantly more spiral ganglion neurons could be preserved in the basal turn of the cochlea [44]. Warnecke et al. showed that a continuous release of BDNF from a particular fibroblast cell line on silicone improved the survival of spiral ganglion cells in vitro and in vivo [65].

A recent in vitro study investigated the possibility of the biological modification of the surfaces of CI electrode carriers with the objective of long-term application of neurotrophins. The group showed that magnetic particles improve the adhesion of a fibroblast cell line [1].

Richardson and coworkers used polypyrrole, an electro-active polymer, in which the growth factor neurotrophin 3 (NT3) was incorporated in order to protect auditory neurons from degeneration after sensorineural hearing loss and to stimulate the growth of neurites to the electrode [45]. Under in vivo conditions, they further found a protective effect on ganglion cells after treatment with aminoglycosides when the inserted electrode carrier was coated with the previously tested polymer incorporating NT3 [46].

In the context of the NANOCI project, a multinational group of researchers worked on the improvement of frequency resolution with reduced energy consumption of future CIs. The “proof of concept” could be provided for a targeted outgrowth of auditory neurons to the stimulation electrodes in vivo and a fivefold reduction in energy used for stimulation in an in vitro set-up [34].

### Antiapoptotic substances

Currently, research is focused on the application of glucocorticosteroids and antiapoptotic substances. These applications are aimed at minimizing fibrosis and hearing loss due to surgical trauma caused by insertion of the CI electrode [11, 19, 63]. The objective is to preserve residual hearing for combined electro-acoustic stimulation (EAS or Hybrid CI).

Preservation of residual hearing allows for combined electro-acoustic stimulation

Eshraghi and coworkers intracochlearly applied the antiapoptotically effective MAPK/JNK pathway inhibitor D‑JNKI-1 continuously over 7 days after implantation of the electrode carrier. In contrast to the control group that underwent surgery without subsequent treatment with the apoptosis inhibitor, a progressive deterioration of the hearing thresholds could thus be avoided [10].

Other potential agents for protection of neuronal structures after insertion trauma are the substances CEP-1347 [40] and SP 600125 [7, 11] also inhibiting the MAPK/JNK signaling pathway. For CEP-1347, a protective effect on hair cells after noise exposure was found [40]. The antiapoptotic agent AM-111 is currently tested in clinical studies on the treatment of acute noise-induced trauma and idiopathic sudden sensorineural hearing loss. It might also be an interesting option for application in combination with cochlear implants [36, 58, 59].

### Glucocorticosteroids

Glucocorticosteroids have manifold effects in the auditory system. An overview was provided by Meltser and Canlon in 2011 [32] and by Trune and Canlon in 2012 [62].

In vitro studies showed, for example, that the loss of auditory sensory cells after exposure to tumor necrosis factor α could be reduced by a dexamethasone-releasing polymer that may be used for coating the electrode carriers [6].

Jolly and coworkers showed in an in vivo set-up that the hearing loss caused by insertion trauma could be significantly reduced after implantation of silicone rods that were loaded with dexamethasone, which was released over a period of several weeks. Drug-free silicone rods were implanted in the control group [21].

Generally, it is possible to apply drugs also independently from the CI electrode carrier, e. g., via a catheter that is temporarily inserted into the cochlea before CI insertion. This catheter may be advanced into the scala tympani and then the drug is very slowly instilled. However, experiments in a cochlear model demonstrated that the drug distributes mostly from the catheter tip in a basal direction because the fluid volume is displaced at the round window. Further distribution of the substance in an apical direction can only occur via diffusion [21].

Takumi and coworkers investigated gene expression patterns after insertion of dexamethasone-releasing CI electrodes in guinea pigs. They found a modification in gene regulation in comparison to drug-free CI electrodes [61].

In a functional, morphological, and pharmacokinetic study, Liu et al. investigated effects of implantation of a dexamethasone-releasing CI electrode. In both groups, the drug-carrying CI electrodes group and the control group (drug-free implants), hearing thresholds deteriorated immediately after surgery. While the control group experienced almost no hearing improvement up to 6 months after surgery, a slow improvement of the hearing thresholds was observed in the group with dexamethasone-loaded CI electrodes within 1–12 weeks [29, 30].

Douchement and coworkers evaluated the effects on hearing with application of conventional electrode carriers and carriers loaded with 1% and 10% dexamethasone. For both concentrations a significantly better hearing preservation was observed 6 weeks after implantation compared with conventional electrode carriers. For assessment of the long-term effect, the hearing thresholds were again measured 1 year later. Hereby, the protective effect on the preservation of the hearing functions could be proven most clearly in the high frequencies [8].

Lower impedances were measured in patients with dexamethasone-eluting CI electrodes

Farhadi and coworkers showed a local immune suppression in the cochlea after electrode insertion trauma by dexamethasone-loaded electrode carriers. In this context, a loading with 2% led to a significantly reduced migration of immune cells into the cochlea [13].

In a recently published study, Bas and coworkers showed that dexamethasone-releasing CI electrode arrays were protective against electrode insertion trauma. Deterioration of hearing threshold, loss of sensory cells, damage of neural elements, increased impedances, and fibrosis could be reduced depending on the applied dosage. For sufficient protection of neural elements, a concentration of at least 1% in the arrays was necessary [3].

Wilk and coworkers confirmed the correlation of increased impedance and fibrosis with cochlear implantation and their reduction by applying dexamethasone-eluting electrode carriers. The most severe fibroses were observed in the basal area of the cochlea near the round window. CI electrodes with dexamethasone concentrations of 1% as well as 10% significantly reduced fibrosis around the electrode array. At 3 months after implantation, the impedances in both groups with dexamethasone-eluting arrays (1% and 10%) were significantly lower compared with the control group. The group with the higher concentration (10%), however, showed stronger effects [66].

Dexamethasone-releasing CI electrodes have also already been applied in humans. In a pilot study, the safety of dexamethasone-eluting electrodes could be shown in a small patient group, and lower impedances were measured in the group of patients with dexamethasone-eluting CI electrodes [5].

## Problems and future questions

The problems and future questions regarding local drug application in combination with CIs include short-term and long-term effectiveness as well as measurable clinical outcome parameters and possible adverse effects.

Adverse effects might be, for example, immunosuppressive effects of corticosteroids or the development of biofilms [20]. Other adverse effects caused by neurotrophins or viral vectors must be investigated and excluded [54].

Regarding effectiveness, it must be considered that, e.g., neurotrophins have only short half-lives, substance reservoirs become empty, and implanted pumping systems are associated with specific technical problems and risks. Cell-based therapies may provide solutions [37]; hereby, cells are stimulated in order to produce neurotrophic factors or release neurotrophic factors after genetic modification [65].

## Clinical case reports

### Case report 1

A 63-year-old patient with bilateral progressive hearing loss since around 1998 received hearing aids in 2003. From 2012, he no longer recognized speech despite hearing aids. Since around 2000, the patient had additionally been suffering from vertigo. In 2009, the patient had an apoplexy. The ENT-specific findings and diagnoses consisted of deafness in the right ear and severe hearing loss on the left side as well as bilateral vestibulopathy due to “degenerative inner ear disease.”

In November 2013, the patient received a CI on the right side (CI24RE(CA), Cochlear Ltd., Australia). The intraoperative electrophysiological tests showed homogeneous and regular impedances with values of 5–10 kOhms at all electrodes. The acoustic reflex could not be provoked. The ECAP values measured by means of neural response telemetry (NRT) were between 205 and 235 current units (cu). The first fitting of the audio processor was successful, so that hearing impressions could be achieved. Hearing developed positively in the course of the fittings and rehabilitation measures.

**Fig. 2:**
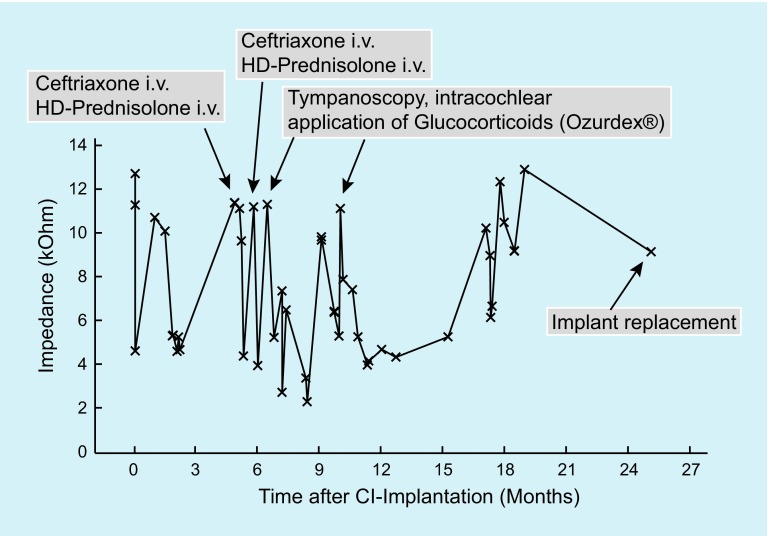
Time course of impedances of a typical cochlear implant (*CI*) electrode (no. 6) together with the times of systemic and local glucocorticoid application in patient 1 from the case reports. *HD* high dose

About 5 months after cochlear implantation on the right side, the patient reported intermittently severe, increasing vertigo and a fluctuating hearing capacity. Microscopy of the ear showed normal findings. However, over all electrodes significantly increased impedances were measured. The patient received intravenous antibiotics with ceftriaxone (1 × 2 g/day for 7 days) and a systemic high-dose prednisolone therapy (250 mg/day for 5 days) leading to an improvement of the complaints and reduction of the impedances (Fig. 2).

One month later, the patient reported increased vertigo with a tendency to fall to the right side. Again, hearing was fluctuating. Over all electrodes the impedances were increased. Clinically, the beginning of fibrosis of the cochlea was suspected. The patient received intravenous antibiotic therapy with ceftriaxone and intravenous systemic high-dose prednisolone therapy followed by an oral therapy with 5 mg prednisolone per day for 2 months leading to improved hearing, normalization of the impedances, and decrease of vertigo.

**Fig. 3:**
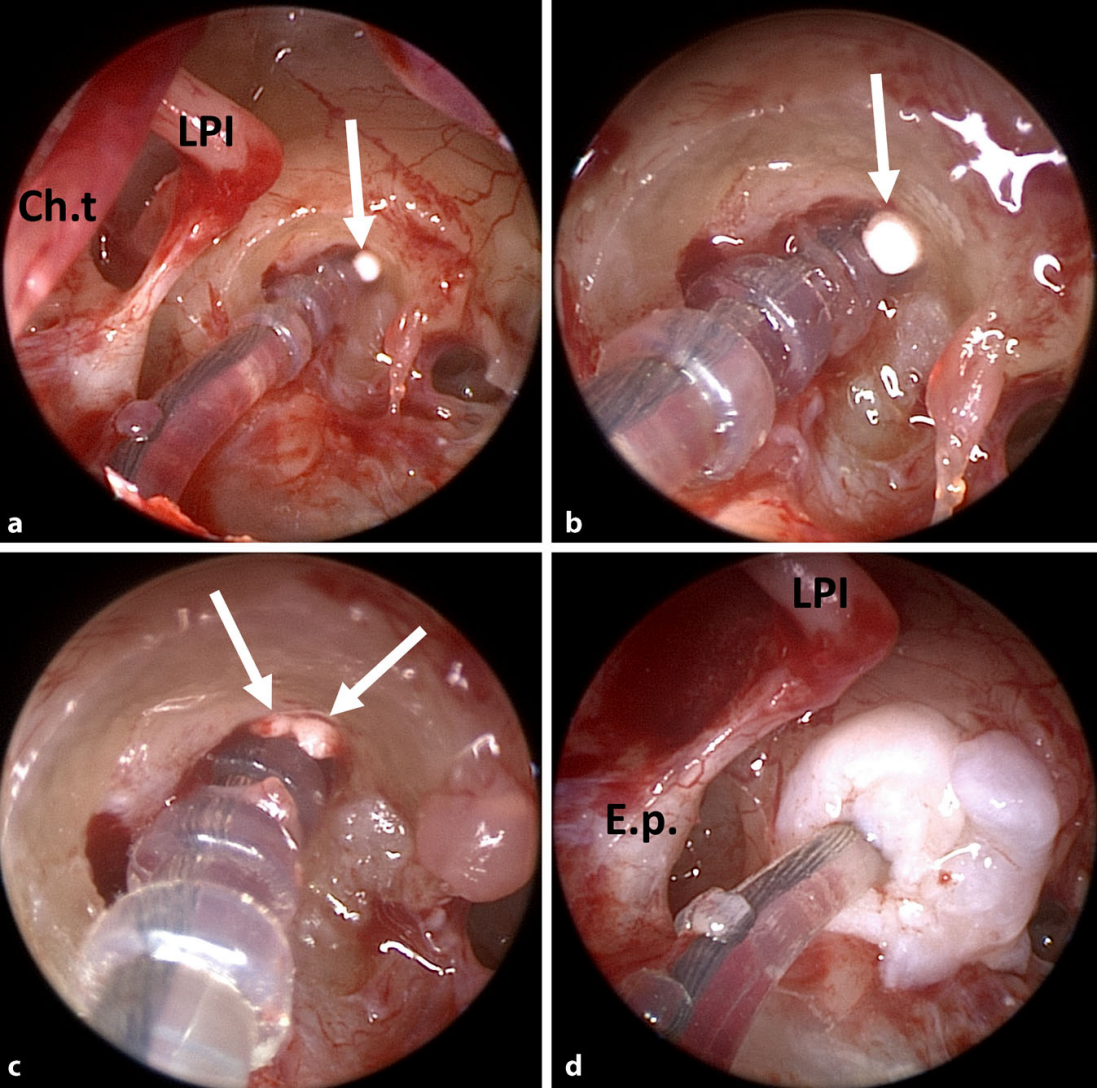
Implantation of single parts (size about 3 mm × 0.46 mm, each) of a biocompatible, degradable drug carrier approved for intravitreal injection (Ozurdex®, *arrows*) in addition to the cochlear implant electrode carrier in the scala tympani in patient 1 (see case reports). **a** Endoscopic view of the middle ear. Insertion of one half (**a, b**) and then of the other half (**c**) of the degradable drug-delivery system. **d** Sealing of the insertion point of the electrode carrier into the scala tympani with connective tissue. *LPI* long process of the incus; *Ch.* *t.* chorda tympani; *E.p.* eminentia pyramidalis

After further increases in vertigo attacks and varying hearing capacity, tympanoscopy and intracochlear glucocorticoid application were performed with implantation of a controlled-release drug-delivery device (Ozurdex®, Allergan Inc., Irvine, Calif., Fig. 3). This biodegradable drug carrier measures 0.46 × 6 mm and contains 0.7 mg of dexamethasone in a polylactic-co-glycolic acid (PLGA) polymer matrix. PLGA is an organic substance based on lactic acid that is easily degraded by the human body. Ozurdex® is approved for intravitreal application for treating macula edema after retinal vein occlusion and noninfectious uveitis. The drug-delivery device has been applied in the ear after acute, severe, and profound idiopathic hearing loss (sudden hearing loss) in the context of secondary or tertiary (“salvage,” “rescue”) therapy at the round window membrane [42]. Additionally, allergy testing of the CI components was performed without any signs of intolerance (regular patch test). After local drug application and a 4‑week pause in the use of the CI, regular impedances were measured.

A biodegradable drug-delivery system was introduced for individual cases

Three months later (in September 2014), vertigo increased again and hearing was again fluctuating. The ear microscopic findings were normal. Over all electrodes, clearly increased impedances were measured. Tympanoscopy was performed, again with intracochlear implantation of the dexamethasone-eluting drug delivery device (Ozurdex®). Additionally, triamcinolone (Volon A, 10 g/ml) on Curaspon® was applied into the oval window niche. To date, the diffusion of glucocorticosteroids through the oval window niche into the scala vestibuli or into the vestibulum has not been investigated experimentally. However, data from other in vivo experiments with gadolinium and gentamycin allow us to assume that glucocorticoids are also taken up through the oval window [22, 53]. A follow-up examination in October 2014 showed homogeneous and reduced impedances, a slightly improved hearing, and no vertigo. In November 2014, the patient started to complain of postural instability. Walking over longer distances was only possible with a walking frame. Again, the impedances were increased over all electrodes. From May 2015, no speech recognition was found on the right side and the impedances have remained increased. Vertigo (vestibulopathy with tendency to fall to the right side) was unchanged. Walking was only possible with an accompanying person or a walking frame.

In June 2015, despite partly regular impedances, no hearing with the CI was possible. An intensive functional test of the implant (“integrity test”) was performed by the manufacturer, which did not show any particularities. ECAPs, however, could not be evoked. Stimulation of the facial nerve with very high amplitudes could not be provoked.

One year after the first CI implantation, no clear fluid signal was detected by magnetic resonance imaging in the basal turn on the right side with the inserted CI electrode. This was interpreted as a possible postinflammatory finding. No signs of an intracranial tumor were found. In comparison with the preoperative examination from 2013, a newly diagnosed lesion of unclear genesis was found with extension from the cerebellar peduncle on the right side to the right nucleus of the trigeminus nerve with involvement of the main nuclear areas of the seventh and eighth cranial nerves. Furthermore, a known, severe cortical siderosis was found.

In December 2015, it was decided to change the CI on the right side. Despite (at that time) absence of vertigo and regular impedances during audio processor fitting, no hearing impression could be achieved.

Fig. 2 depicts the course of the electrode impedances of an exemplary CI electrode as well as medical and surgical interventions.

### Case report 2

A 62-year-old female patient suffered from bilateral progressive hearing loss and had received hearing aids in both ears (word recognition score of monosyllables with hearing aids at 65 dB SPL on the right: 55% and on the left: 45%). As secondary diagnosis, only arterial hypertension was known.

In November 2014, the patient received a CI on the right side (CI 422, Cochlear Ltd., Australia). The intraoperative electrophysiological testing showed regular impedances over all electrodes at a level of 13.3–16.1 kOhm. The acoustic reflex could be provoked. On all electrodes, ECAPs could be evoked by NRT and with 154–252 cu.

Three months after cochlear implantation, postural instability with vestibulopathy was found without changes in hearing capacity with the CI but with increased impedances. The patient received intravenous antibiotics of cefuroxime (3 × 1.5 g/day) as well as intravenous systemic high-dose prednisolone (250 mg/day for 5 days). The patient’s vertigo complaints reduced and the impedances decreased.

In May 2015 (6 months after cochlear implantation), the patient complained of rotary vertigo, with unchanged hearing with the CI. An ENT examination yielded regular postoperative findings; the impedances were inhomogeneous but increased. The patient received intravenous high-dose prednisolone (250 mg/day for 5 days), followed by an oral taper with continuous dose reduction over 16 days. This therapy led to a reduction in the impedances and improvement of the vertigo. However, as early as in June 2015, rotary vertigo attacks occurred again accompanied by nausea and vomiting so that inpatient treatment with high-dose prednisolone and with ceftriaxone was initiated. This led to quick improvement of the complaints, and thus continuous application of dexamethasone by means of an implantable drug carrier on the right side was discussed with the patient. Tympanoscopy was performed to exclude a labyrinth fistula in the area of the entry of the CI electrode into the scala tympani (via the round window niche) and the aforementioned drug-delivery device for continuous dexamethasone release (Ozurdex®) was implanted into the basal turn of scala tympani next to the CI electrode carrier.

In August 2015, rotary vertigo, increasing impedances, and deterioration of hearing with CI led to tympanoscopy on the right side with atticotomy, removal of the connective tissue adhesions in the oval window niche, and the application of gentamycin on Curaspon® into the oval window niche. Already on the first postoperative day, vertigo attacks ceased. However, 2 weeks after surgery, vertigo was observed again along with deterioration in hearing.

After increasing vertigo (vestibulopathy, postural instability), and further hearing loss with CI, transtympanic stapedectomy was performed in October 2015 with destruction of the labyrinth (mechanically and with insertion of gentamycin on Curaspon® into the vestibulum). Since then, the patient is completely free of vertigo complaints and hearing with the CI is good. The patient asked for implantation of a CI on the contralateral side because hearing on that side had further deteriorated.

**Fig. 4:**
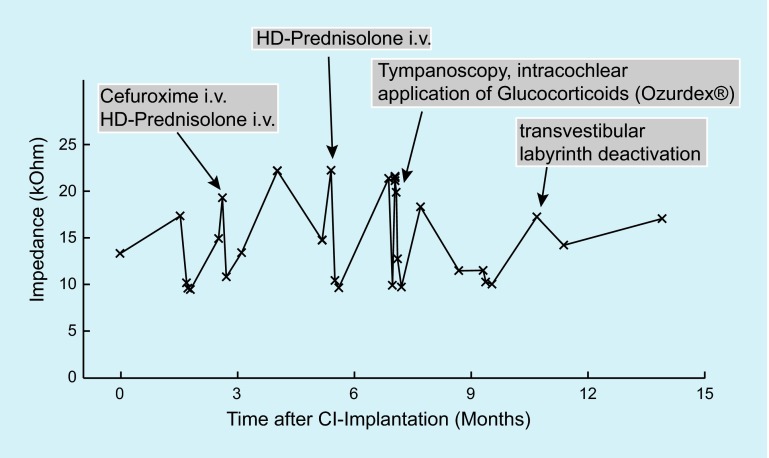
Time course of the impedances of a typical cochlear implant (*CI*) electrode (no. 9) together with the times of medical and surgical interventions in patient 2 (see case reports). *HD* high dose

Fig. 4 shows the course of the electrode impedances of an exemplary CI electrode as well as medical and surgical interventions.

## Stimulation of intracochlear drug concentrations

**Fig. 5:**
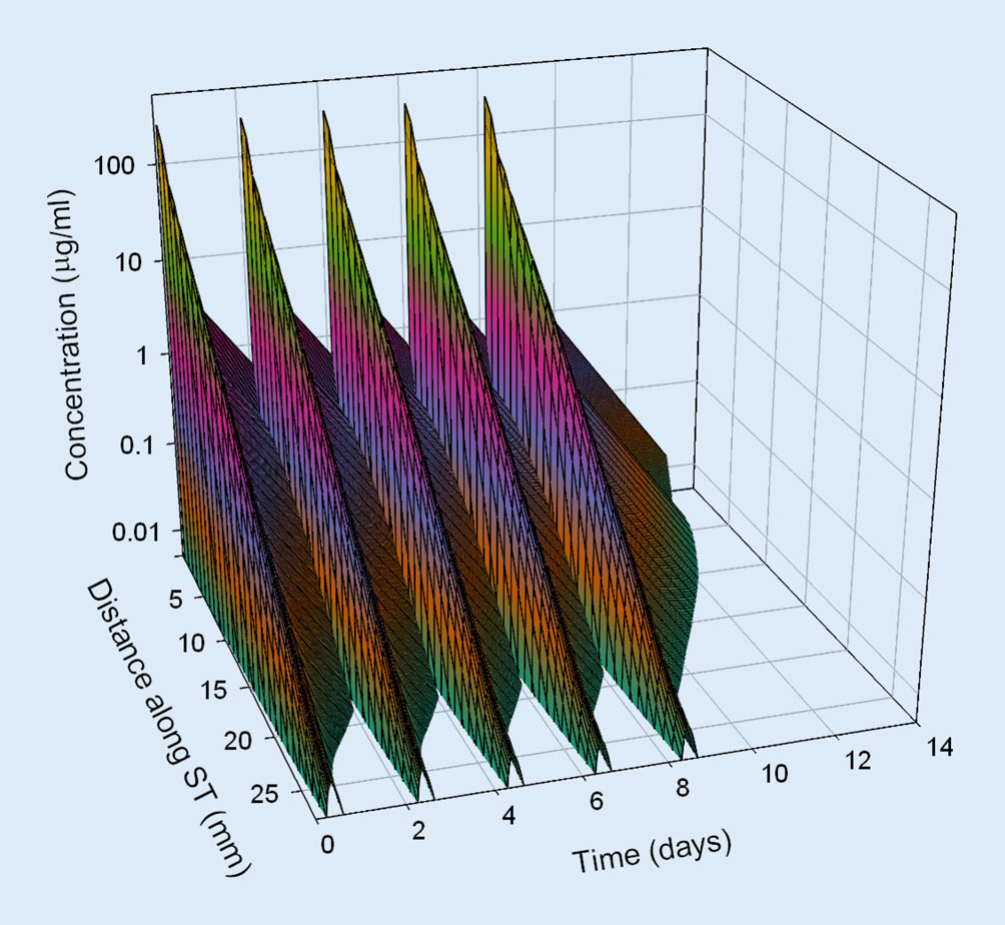
Calculated concentration in the perilymph for intratympanic injection (extracochlear application) of dexamethasone phosphate solution (1× per day, every 2 days, total of 5 injections). *ST* scala tympani

**Fig. 6:**
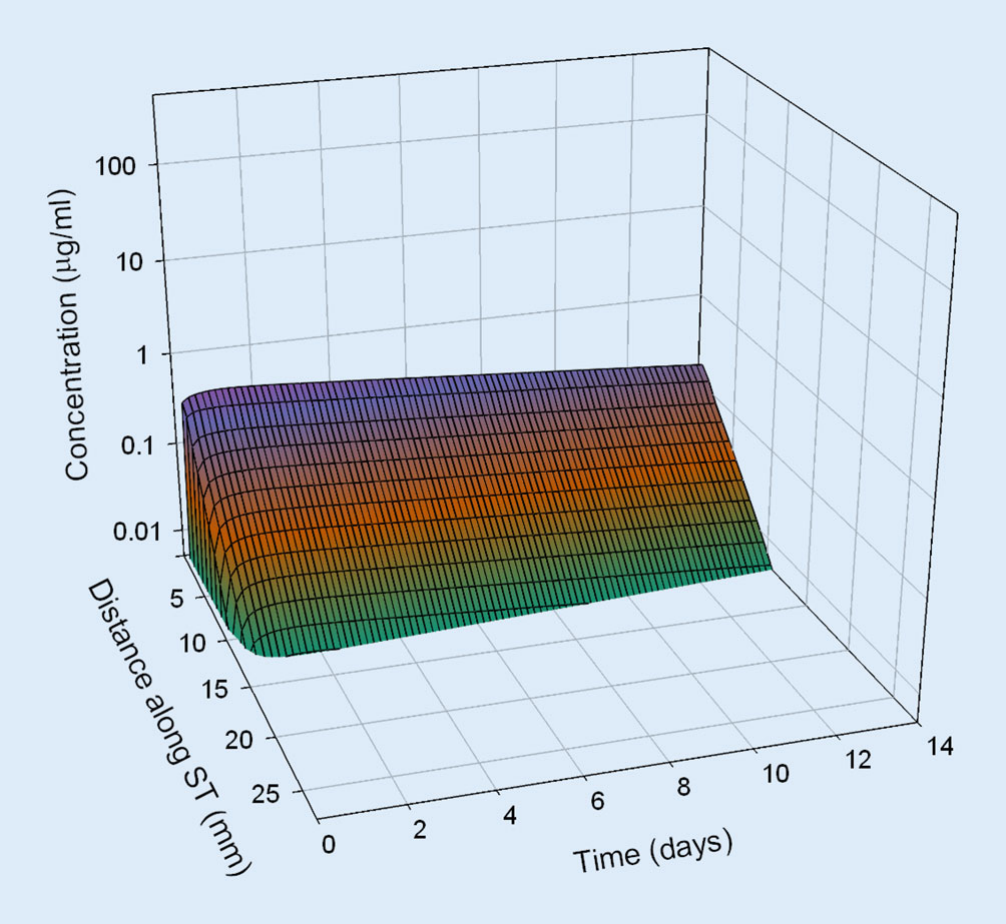
Calculated concentration of dexamethasone in the perilymph for extracochlear application of Ozurdex® to the round window membrane. *ST* scala tympani

**Fig. 7:**
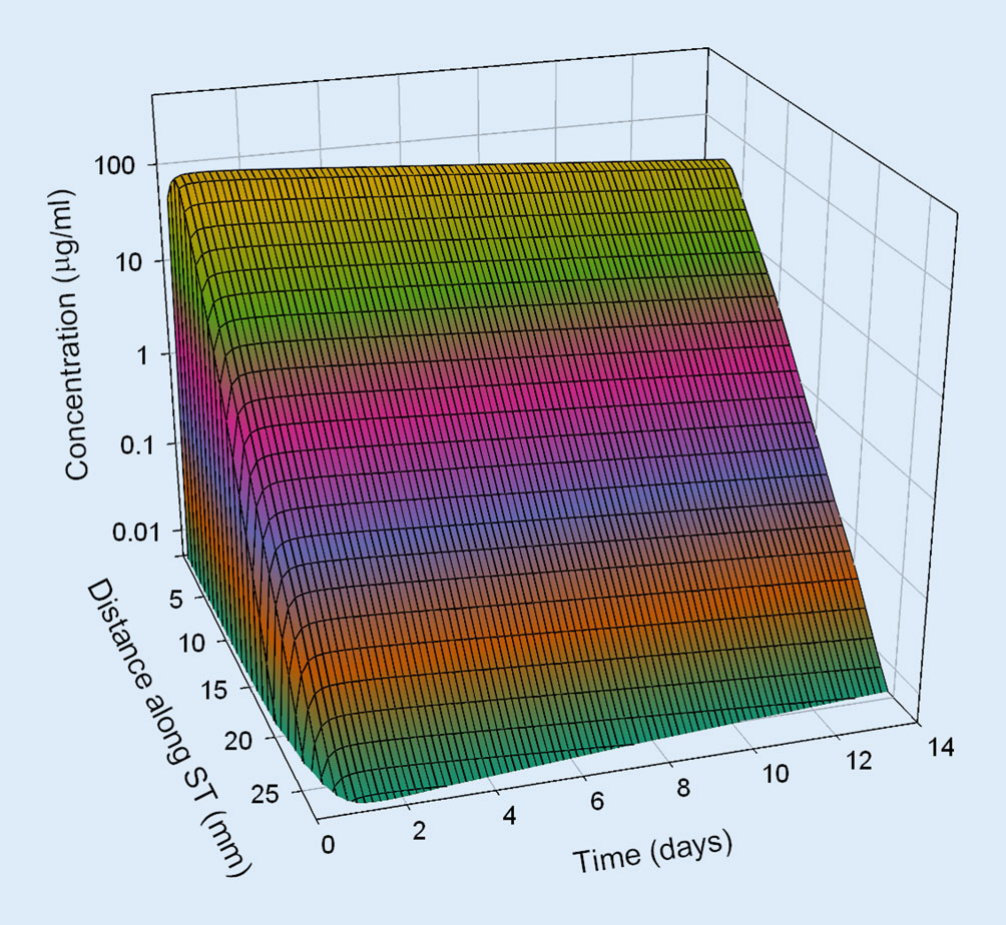
Calculated concentration of dexamethasone in the perilymph for intracochlear application of Ozurdex® in addition to a cochlear implant electrode carrier (Nucleus CI24RE(CA)) in the basal part of the basal turn of the scala tympani (case report 1). *ST* scala tympani

**Fig. 8:**
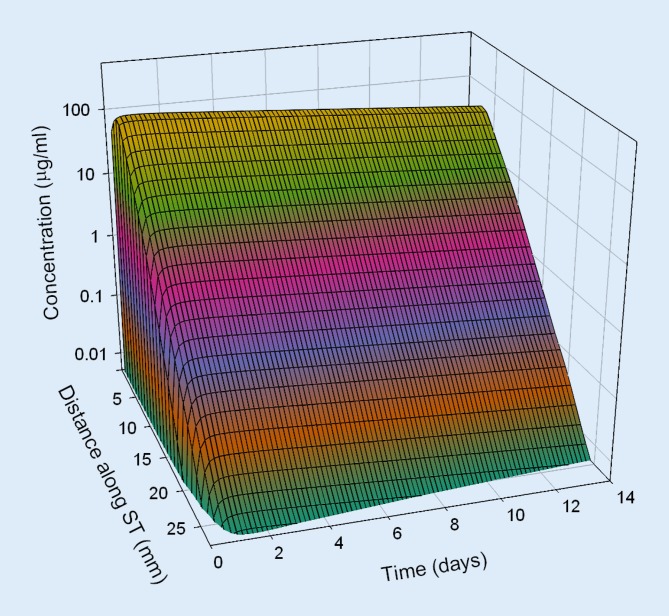
Calculated concentration of dexamethasone in the perilymph for intracochlear application of Ozurdex® in addition to a cochlear implant electrode carrier (Nucleus CI422) in the basal part of the basal turn of the scala tympani (case report 2). *ST* scala tympani

Based on pharmacokinetic data from in vivo investigations in guinea pigs, dexamethasone concentration in the perilymph of the scala tympani was estimated for different application strategies in humans by means of a validated computer model for calculation of substance concentrations in inner ear fluids (FluidSim v3.1; http://otocore.wustl.edu/saltlab/). Simulated application strategies were:

- Repeated intratympanic injection of dexamethasone phosphate solution (4 mg/ml, 0.3 ml; 30 min) into the middle ear (once every 2 days, total of five injections), (Fig. 5).
- Extracochlear application of an absorbable PLGA-based drug carrier with continuous elution of a total of 0.7 mg dexamethasone for about 8 weeks (Ozurdex®) in the round window niche (Fig. 6).
- Intracochlear application of Ozurdex® in the basal parts of the basal turn of the scala tympani in addition to an already implanted CI electrode carrier (case report 1: Figs. 2, 3 and 7; case report 2: Figs. 4 and 8).

The pharmacokinetic parameters for dexamethasone were based on previous investigations [52] and re-analysis of published experimental data from animal experiments in guinea pigs [51]. For the dexamethasone phosphate injections, a round window permeability of 76 * 10^−9^ m²/s, a diffusion coefficient of 0.72 * 10^−9^ m²/s, and an elimination half-time from the scala tympani of 18.3 min were assumed. For the free dexamethasone base (Ozurdex®), a round window permeability of 2.37 * 10^−9^ m²/s, a diffusion coefficient of 0.77 * 10^−9^ m²/s, and an elimination half-time of 22.5 min were used. For the elution rate from the drug-delivery device, Ozurdex®, an exponential decrease with a half-time of 10 days was assumed.

Continuous intracochlear application led to higher drug levels along the scala tympani

The continuous intracochlear application resulted in a stable concentration for several weeks (only the first 2 weeks are shown), with higher drug levels more apically along the scala tympani. Longitudinal concentration gradients remained because of the rapid elimination of dexamethasone (elimination half-time: 22.5 min [52]).

## Conclusion for clinical practice

- Beside the (“off-label”) strategy of local drug application to the inner ear by means of intratympanic injection that is already established in the clinic, the focus of current research is increasingly directed toward intracochlear application, for which the first clinical trials are already underway.
- Intracochlear drug application in the context of cochlear implantation has the advantage that the cochlea is already opened owing to insertion of the CI electrode carrier.
- In the future, intracochlear application could also play a role for indications other than hearing rehabilitation with CI, since it appears to be necessary for certain therapeutic strategies, especially gene- and cell-based therapy of the inner ear.
